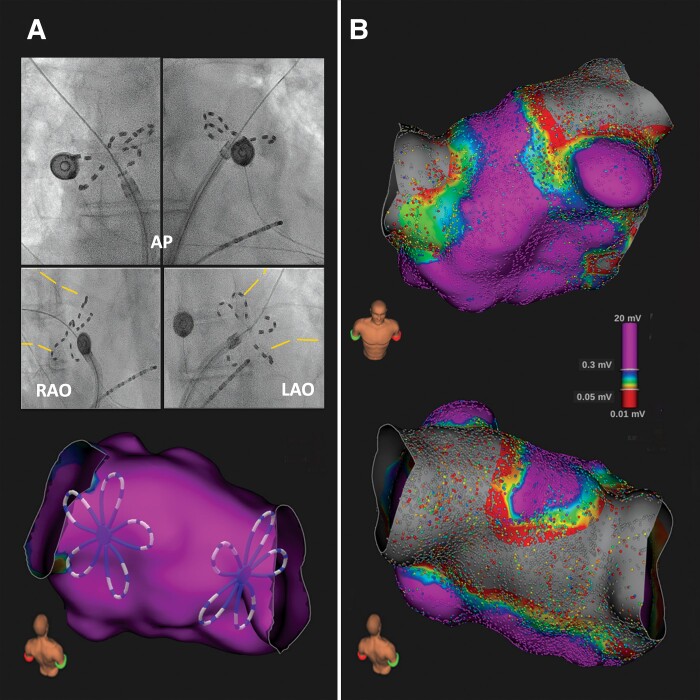# Unexpected fused posterior wall lesions after pulsed-field pulmonary vein isolation

**DOI:** 10.1093/europace/euac113

**Published:** 2022-12-02

**Authors:** Vincenzo Miraglia, Felicia Lipartiti, Alvise Del Monte, Gian-Battista Chierchia, Carlo de Asmundis, Erwin Ströker

**Affiliations:** Heart Rhythm Management Centre, Postgraduate Course in Cardiac Electrophysiology and Pacing, European Reference Networks Guard-Heart, Vrije Universiteit Brussel, Universitair Ziekenhuis Brussel, Brussels, Belgium; Heart Rhythm Management Centre, Postgraduate Course in Cardiac Electrophysiology and Pacing, European Reference Networks Guard-Heart, Vrije Universiteit Brussel, Universitair Ziekenhuis Brussel, Brussels, Belgium; Heart Rhythm Management Centre, Postgraduate Course in Cardiac Electrophysiology and Pacing, European Reference Networks Guard-Heart, Vrije Universiteit Brussel, Universitair Ziekenhuis Brussel, Brussels, Belgium; Heart Rhythm Management Centre, Postgraduate Course in Cardiac Electrophysiology and Pacing, European Reference Networks Guard-Heart, Vrije Universiteit Brussel, Universitair Ziekenhuis Brussel, Brussels, Belgium; Heart Rhythm Management Centre, Postgraduate Course in Cardiac Electrophysiology and Pacing, European Reference Networks Guard-Heart, Vrije Universiteit Brussel, Universitair Ziekenhuis Brussel, Brussels, Belgium; Heart Rhythm Management Centre, Postgraduate Course in Cardiac Electrophysiology and Pacing, European Reference Networks Guard-Heart, Vrije Universiteit Brussel, Universitair Ziekenhuis Brussel, Brussels, Belgium

We present the case of a 55-year-old patient with paroxysmal atrial fibrillation referred for pulmonary vein isolation (PVI) by means of pulsed-field ablation (PFA). Based on preprocedural imaging (computed tomography) showing a bilateral common ostium (CO) as PV variant, the larger penta-spline PFA catheter measuring 35 mm was selected for ablation. Bilaterally, acute PVI was observed during the first applications at the CO wiring the superior branches. Overall, eight application pairs were delivered with four pairs per CO (two pairs wiring superior and inferior branches, respectively, ‘basket’ and ‘flower petal’ deployment pose per pair). Pre- and post-ablation 3D ultra-high-density voltage map of the left atrium was acquired with a 64-pole basket mapping catheter (Orion). The post-ablation map confirmed acute PVI, but showed unexpected fused lesions on the lower posterior wall (PW) in addition (*Panel B*). Although the PFA applications were intentionally delivered for PVI, the finding of extended PW lesions may be explained by the larger sized PFA catheter, the relatively shorter posterior inter-venal distance (although still 39 mm), but clearly also by the unique property of PF technology to obtain tissue lesions not necessarily through high catheter contact, but also in the proximity of the tissue–electrodes interface.

The full-length version of this report can be viewed at: https://www.escardio.org/Education/E-Learning/Clinical-cases/Electrophysiology.

**Figure:**